# Effects of Pharyngeal Electrical Stimulation on Swallow Timings, Clearance and Safety in Post-Stroke Dysphagia: Analysis from the Swallowing Treatment Using Electrical Pharyngeal Stimulation (STEPS) Trial

**DOI:** 10.1155/2021/5520657

**Published:** 2021-06-07

**Authors:** Lisa F. Everton, Jacqueline K. Benfield, Emilia Michou, Shaheen Hamdy, Philip M. Bath

**Affiliations:** ^1^Stroke Trials Unit, Mental Health and Division of Clinical Neuroscience, University of Nottingham, Nottingham, UK; ^2^Speech and Language Therapy, Nottinghamshire Healthcare NHS Foundation Trust, Nottingham, UK; ^3^Vascular Medicine, Division of Medical Sciences and GEM, University of Nottingham, Royal Derby Hospital Centre, Derby, UK; ^4^GI Sciences, Division of Diabetes, Endocrinology and Gastroenterology, School of Medicine Sciences, University of Manchester and the Manchester Academic Health Sciences Centre, UK; ^5^Speech and Language Therapy Department, School of Rehabilitation Sciences, University of Patras, Patras, Greece; ^6^Stroke, Nottingham University Hospitals NHS Trust, Nottingham, UK

## Abstract

Swallowing impairment (dysphagia) post-stroke results in poorer outcomes. Pharyngeal electrical stimulation (PES) is a potential treatment for post-stroke dysphagia. In a post hoc analysis, we investigated PES using videofluoroscopy swallow studies (VFSS) from the STEPS trial incorporating multiple measures of safety (penetration aspiration scale-PAS), speed and duration (timing), and efficiency (clearance), as opposed to the original trial which only measured PAS scores. 81 randomised participants (PES (*N* = 43) versus sham (*N* = 38)) were analysed at baseline and 2 weeks. Participants swallowed up to 6 × 5 ml and 1 × 50 ml of thin liquid barium at 40% *w*/*v*, images at ≥25 fps. Based on PAS, the 5 ml mode bolus (most frequently occurring PAS from 6 × 5 ml) and the worst 50 ml bolus were chosen for further analysis. Eight timing measures were performed, including stage transition duration (STD) and pharyngeal transit time (PTT). Clearance measures comprised oral and pharyngeal residue and swallows to clear. Comparisons of change of scoring outcomes between PES and sham were done at 2 weeks. Wilcoxon Signed Ranks Test was also used to evaluate longitudinal changes from both groups' combined results at two weeks. Between-group analysis showed no statistically significant differences. Issues with suboptimal image quality and frame rate acquisition affected final numbers. At two weeks, both groups demonstrated a significant improvement in most safety scores (PAS) and STD, possibly due to spontaneous recovery or a combination of spontaneous recovery and swallowing treatment and usual care. A nonsignificant trend for improvement was seen in other timing measures, including PTT. This study, which conducted additional measurements of kinematic and residue analysis on the STEPS data did not detect “missed” improvements in swallowing function that the PAS is not designed to measure. However, more studies with greater numbers are required.

## 1. Introduction

Dysphagia is associated with poorer outcomes [1, 2]. Despite this, there are few proven treatments for post-stroke (PSD) dysphagia and a pressing need for more high-quality trials in order to assess which specific interventions are effective [3]. Swallowing is a highly complex bodily function comprising multiple components of extremely precise, rapid, and often overlapping events, which is frequently assessed using the Penetration Aspiration Scale (PAS) [4]. However, the PAS is complicated by high intrasubject variability [5–7], a lack of standardised analysis methods to apply to PAS scores and variability in statistical methods used to analyse the chosen scores [6, 8]. Furthermore, the PAS only measures one aspect of swallowing, i.e., the direction of bolus flow, and does not consider collective measures of speed and duration (bolus timing) and efficiency (bolus clearance) [9]. Using multiple measures of swallowing may provide a more complete measure of swallowing function [9, 10]. Pharyngeal electrical stimulation (PES) is a potential treatment for PSD and has been used in several published studies to date [7, 11–13]. The largest of these was the Swallowing Treatment using Electrical Pharyngeal Stimulation Trial (STEPS) [7]. This trial only compared swallowing safety using PAS. We therefore investigated the effect of PES on multiple measures of both speed and duration (timing) and efficiency (clearance) as well as safety (PAS) by conducting a retrospective analysis on the STEPS data.

## 2. Materials and Methods

### 2.1. Participants and Videofluoroscopy Swallowing Studies

Data from the STEPS trial was used to carry out the analysis [7]. In brief, participants (stroke onset within 42 days) were given up to 7 boli (comprising 6 × 5 ml boli and 1 × 50 ml (cup drinking) bolus) of thin fluids at 40% wt/vol of either Omnipaque 300, Visipaque 270, or Accupaque during a baseline VFSS. If consecutive occurrences of aspiration were seen, “stop” criteria were applied; hence, not all participants received all 7 boli. Participants meeting the recruitment criteria (any bolus with PAS >2) were then randomly assigned to either PES or sham treatment, which was given for 3 consecutive days. A follow-up VFSS was conducted at weeks 2 and 12 according to the STEPS protocol. The primary outcome was the reduction in the mean PAS score of all boli between baseline and VFSS at 2 weeks. Eighteen hospital sites participated in the STEPS study. In the current study, participants who were randomised to the active (PES) group or sham group and who had a baseline, and two-week VFSS were included, as well as those with VFSS data recorded at a frame rate of ≥25 fps. Files were viewed in Quick Time 7 (Apple Inc, USA) using frame-by-frame analysis. Data was analysed on several different measurements, and these are summarised in supplementary table 1 (in the Supplementary Material) and detailed below.

### 2.2. PAS, Timing, and Clearance Measures

Every swallow performed to clear each 6 × 5 ml bolus (i.e., primary and secondary (clearing swallows)) was given a PAS score. The highest PAS score from each 5 ml bolus was identified, resulting in 6 PAS scores. Of these, the mode PAS, i.e., the PAS score occurring most frequently across the 6 boli [6], was chosen for further analysis. For the 50 ml bolus, the worst PAS score was chosen for analysis. Several timing measures were performed on the 5 ml mode bolus: global oral transit time (gOTT) as defined by the author for this study, stage transition duration (STD) [14], initiation of laryngeal closure (ILC) [10], laryngeal vestibule closure reaction time (LVCrt) [15], laryngeal closure duration (LCD) [16], pharyngeal response time (PRT) [16], pharyngeal transit time (PTT) [17], and upper oesophageal sphincter duration (UOSD) [17, 18]. For the mode 5 ml bolus and the worst 50 ml bolus, the Modified Barium Swallowing Impairment Profile (MBSImP) [19] was also used to score initiation of the pharyngeal swallow (component 6). For both boli, clearance measures comprising oral and pharyngeal residue (components 5 and 16 of the MBSImP), and a number of swallows to clear were also measured. Operational definitions of the timing measures used in this study are listed in supplementary table 2. Timing and clearance measures were always taken from the primary swallow (including those swallows showing secondary aspiration). The mean PAS score across all 5 ml boli and the mean 50 ml bolus was also calculated for comparison.

### 2.3. Ethics

The study underpinning the data had national ethics approvals and patients (or surrogates) had given written informed consent. The trial was registered as ISRCTN25681641.

### 2.4. Statistical Analysis

Baseline characteristics and baseline VFSS measures of participants were determined using descriptive statistics. The Chi-Square test and Fisher's Exact Probability Test (binary and ordinal variables) and Welch's *T*-Test (continuous variables) were used to test for significant differences between the groups at baseline. The primary outcome was safety, timing, and clearance measures of the 5 ml mode bolus, and the secondary outcome was the same measures for the worst 50 ml bolus. The change score from baseline to two weeks for each group (PES versus sham) was calculated and compared using the Independent *T*-Test (unequal variances assumed) for timing (continuous) measures and the Mann–Whitney *U* Test for clearance (ordinal) measures. Wilcoxon Signed Ranks Test was used to compare both groups' combined results at two weeks to evaluate longitudinal changes.

### 2.5. Reliability Testing

Details of reliability information for the safety, timing, and clearance measures used in this study have been submitted for publication.

## 3. Results

### 3.1. Participants and Videofluoroscopy Swallowing Studies

From the original dataset, 126 (of 162) participants received PES or sham and had both a VFSS completed at baseline and 2 weeks. This group comprised the primary outcome population in the STEPS study and was also used for the current study. Further analysis revealed that 42 participants in this group had VFSS data recorded at a frame rate <25 fps, two files were missing, and one file was unanalysable. These files were excluded from this study (the excluded group). This resulted in a final total of 81 files (64.3%) from the 126 participants in the timings and clearance analysis for this study (the included group). In this included group, 71 files (88%) were recorded at a frame rate of 25 fps and 10 files (12%) were recorded at 30 fps. Supplementary figure 1 details the breakdown of frame rates for all files (*N* = 126).

Table 1 details the baseline characteristics of the excluded group (*N* = 45) versus the included group (*N* = 81). No significant differences were apparent between these groups at baseline, except for ethnicity. Table 1 also details the baseline characteristics of the included group (*N* = 81), according to PES (*N* = 43) and sham (*N* = 38). At baseline, the sham group was more dependant (modified Rankin Score) and more disabled (Barthel Index). However, clinical dysphagia severity (Dysphagia Severity Rating Scale) [20] and feeding route were similar between the groups.

Fifteen hospital sites (supplementary figure 2) were included in the study. Most sites contributed <10% of the overall data whilst one site contributed 37% of data. A significant amount of measures were unable to be calculated due to poor imaging quality and on occasions, reduced field of view. This affected the final numbers for statistical analysis as calculating the change score required data to be available from both baseline and two-week timepoints. In addition, as each component comprised two measures at each timepoint (for example, STD comprised measures for bolus head at ramus and hyoid onset), for a dataset to be complete, four measures were required in total.

### 3.2. PAS, Timing, and Clearance Measures

When comparing outcomes between the PES versus sham group, at baseline for the modal bolus (Table 2), there were no significant differences except for LCD (*P* = 0.039) and number of secondary swallows (*P* = 0.048). When comparing the change score at two weeks (Table 3), there were no significant differences between the groups for any measures. For the worst 50 ml bolus, no significant differences were seen between the groups at baseline (Table 4) or for the two-week score change for any measures (Table 5).

When comparing outcomes of both groups (supplementary figure 3) combined at baseline and two weeks (to assess for longitudinal changes), significant improvements in safety scores were seen in all PAS conditions (i.e., mode 5 ml bolus, mean of 5ml boli, and worst 50 ml bolus) with the exception of the mean 50 ml PAS. For measures of timing (supplementary figure 4), the only measure of speed which showed a significant improvement was STD (*Z* = −2.058, *P* < 0.04). However, there was a nonsignificant trend for improved scores for OTT, ILC, and PTT at two weeks but not for LVCrt and PRT. Minimal changes in duration measures (LCD and UOSD) were seen. Large standard deviations were seen at baseline for some timing measures due to the inclusion of a small number of extreme outliers. However, this data was not omitted because it was felt that these scores represent a minority of severe patients that are seen in acute stroke, who do present with very slow swallowing function. As such, this data contains important information which should not be discarded [21]. In addition, the statistical method used with this data, Wilcoxon Signed Ranks Test, is an appropriate test to use with outliers. With this test, each value is ranked in order and then the sums are ranked and hence extreme values do not heavily influence the sum [21].

There were no significant changes for IPS or any clearance measures; (supplementary figure 5) although, there was a trend for less swallows at two weeks in the 50 ml bolus.

## 4. Discussion

### 4.1. PAS, Timing, and Clearance Measures

This current study only included imaging data from the original STEPS trial at ≥25 fps. Our findings with regards to PA scores agree with the overall conclusion from the original STEPS study which also did not show a significant change between groups for safety. Furthermore, this current study which conducted additional temporal and clearance measures on this data has not demonstrated any new or “undetected” significant differences between the groups. In other acute stroke treatment studies using PES, improvements in safety (PAS scores) were seen in smaller studies [12, 13] but not in the larger multicentre STEPS Trial from which a portion of the data in the current study is taken from [7]. In chronic stroke patients, improvements in PAS scores post-PES were seen in one study [22]. Improvements in timing measures, namely, STD and PTT, were reported in one previous study in acute stroke patients, using PES [11], although a further study also using PES in acute stroke patients reported no improvements in these two measures or for OTT or LCD [12]. With regard to clearance measures, no studies have reported on the effects of PES using this component.

The significant improvement in PAS scores observed in both groups at two weeks could have been due to spontaneous recovery. Longitudinal observational studies at 1-month post-onset [9] and up to a year [23], as well as treatment studies [24] in acute and subacute stroke, have reported improved PAS scores over time. These clinical findings correlate with physiological evidence from TMS studies demonstrating improved swallow function in many acute stroke patients with dysphagia [25, 26]. It is also possible that the improvement in PAS scores in both groups was due to a combination of spontaneous recovery and/or swallowing treatment (PES and/usual SLT care). This is because, in the original STEPS trial, both groups received usual SLT care, and in order to preserve blinding as much as possible, both groups also received some PES to obtain threshold and tolerance levels [7]. In addition, levels of PES given to participants in the active group may have been at suboptimal levels, so-called “undertreatment” [7] which may account for why no treatment effect was evident in the STEPS trial and in this research.

We chose the mode bolus as the primary method of analysis as it represents the most frequently occurring swallow pattern across a series of swallows. It therefore may be a more instructive way to measure PAS scores, being more representative of a patient's unique swallow pattern, as opposed to the mean or median [6]. Improvements in the mode bolus therefore may be important. Choosing different boli to analyse may also help to inform methods in evaluating the PAS. It is well known that in acute stroke, there is high intrasubject variability of PAS scores, observed both in this study and documented previously [4–7, 24] with some patients showing a worse or better pattern of swallows even across the same bolus [6, 24] and between boli. This makes the task of predicting aspiration challenging [27] and presents challenges for researchers when considering which boli to analyse and how. Future studies with more participants will provide more information either way, both for clinicians and researchers alike.

As with PAS scores, in the acute phase of stroke, one would expect a trend for longitudinal improvements in timings as patients recover [9, 23]. This was also observed in this study and, like the PAS scores, may have been due to a combination of spontaneous recovery and/or usual SLT care and/or PES. The significant change seen in STD alone could be due to increased numbers available for that measure (55) compared to less numbers for OTT (29), ILC (49), and PTT (25). Two-timing measures (LVCrt and PRT) and both duration measures (UOSD and LCD) did not show a trend for improvement. Due to the modest sample size, it is too early to draw any conclusions from these data at present.

No significant results were seen between the groups for the worst 50 ml bolus. Few studies have reported on swallowing larger boli (≥50 ml) in acute stroke patients, perhaps due to concerns regarding safety and one study that did evaluate swallowing of 100 ml of thin barium only included milder patients [28]. No studies involving PES have included a 50 ml bolus, apart from the STEPS study. In the current study, although the 50 ml bolus was swallowed using multiple swallows, it was viewed as one swallowing task (participants were effectively asked to swallow the bolus “in one go”). Hence, the highest (worst) PAS score from this task was chosen to be analysed. This was different to the 5 ml boli which were six separate, discrete swallowing tasks where a mode could be clearly extracted from across the series.

The numerous measures performed in this research represent a comprehensive analysis of swallow safety, timings, and clearance. The findings from this study suggest that including timings and clearance measures did not result in the detection of differences in swallow function that may have been missed using the PAS scale alone. Equally, in the numbers studied, it is probable that there was no effect to be detected, and hence, it is premature to conclude that only using the PAS is enough to capture change in swallowing function following an intervention. The main reason for lower numbers was due to reduced imaging quality and suboptimal frame rate in the final analysis. This has highlighted the importance of acquiring images at the correct frame rate and optimising data quality and field of view as much as possible.

This study has several strengths. These include analysis of a large dataset with deep phenotypic information from a high-fidelity phase III trial that followed a published protocol; a comprehensive analysis which encompasses all aspects of the swallow (safety, speed, duration, and efficiency) and is, to our knowledge, the first study to publish results of stroke patients focusing on the PAS scores from the mode swallow.

However, some limitations are present. As this real-life study included VFSS from 18 different hospitals in 5 countries inevitably, some images were of suboptimal quality, resulting in missing data as image quality was not good enough to allow measurements or were out of field of view. VFSS frame rates also varied both within and between sites which also reduced the number of files available for analysis. In addition, one site contributed significantly more data than other sites which may represent a bias within the results.

In summary, including measures of timing and clearance (in addition to safety measures) did not detect any further changes in swallowing function. Adequately powered studies, assessing the effect of PES in the acute stroke where PES is given solely to the PES group and at the optimal dose (preventing “undertreatment”), are required.

## Figures and Tables

**Table 1:** Baseline characteristics for participants in STEPS: excluded vs. included (*N* = 126); included (*N* = 81, PES vs. sham). Data are number (%), median [interquartile range], or mean (standard deviation). Comparison by Chi-Square (Exact) Test/Fisher's Exact Test (binary/ordinal variables) or Welch's *T*-Test (continuous variables).

	*N*	All	Excluded	Included	*p*	*N*	PES	Sham
Patients	126		45	81		81	43	38
Age, *y*	126	73.4 (11.4)	73.3 (11.8)	73.4 (11.2)	0.98	81	72.8 (10.0)	74.1 (12.5)
Sex, female (%)	126	49 (38.9)	15 (33.3)	34 (42.0)	0.45	81	21 (48.8)	13 (34.2)
Ethnicity, white (%)	126	108 (85.7)	43 (95.6)	65 (80.2)	**0.031**	81	39 (90.7)	36 (94.7)
Modified Rankin Scale (/6)	126	4.1 (1.0)	4.0 (1.1)	4.1 (1.0)	0.47	81	3.9 (1.0)	4.4 (0.9)
Barthel Index (/100)	126	28.9 (29.8)	31.4 (30.8)	27.4 (29.4)	0.48	81	33.8 (32.7)	20.1 (23.4)
Stroke			3 (6.7)	12 (14.8)	0.25	81	43	37
Previous (%)	126	15 (11.9)					9 (20.9)	3 (7.9)
Type, ischaemic (%)	110	96 (87.3)	32 (86.5)	64 (87.7)	1.00	73	33 (86.8)	31 (88.6)
Side of CT lesion (%)	123				0.25	80	43	37
Left		55 (44.7)	20 (46.5)	35 (43.8)			19 (44.2)	16 (43.2)
Right		50 (40.7)	14 (32.6)	36 (45.0)			18 (41.9)	18 (48.6)
No lesion		18 (14.6)	9 (20.9)	9 (11.3)			6 (14.0)	3 (8.1)
Syndrome (%)	126				0.31	81		
TACS		35 (27.8)	13 (28.9)	22 (27.2)			11 (25.6)	11 (28.9)
PACS		49 (38.9)	14 (31.1)	35 (43.2)			21 (48.8)	14 (36.8)
Lacunar		41 (32.5)	17 (37.8)	24 (29.6)			11 (25.6)	13 (34.2)
POCS		1 (0.8)	1 (2.2)	0 (0)			0 (0)	0 (0)
Severity, NIHSS (/42)	126	10.1 (6.5)	9.5 (7.3)	10.4 (6.0)	0.48	81	10.1 (6.1)	10.8 (5.9)
Dysphasia, NIHSS (%)	126	44 (34.9)	13 (28.9)	31 (38.3)	0.33	81	17 (39.5)	14 (36.8)
Onset to randomisation (days) mean (SD)	126	16.2 (9.9)	15.2 (8.3)	16.8 (10.7)	0.33	81	15.4 (10.3)	18.4 (11.1)
Median [IQR]		14 [11]	15.0 [12]	14.0 [16]			15.5 [15]	13.0 [13]
DSRS (/12)	126	7.4 (3.7)	7.8 (3.7)	7.1 (3.7)	0.35	81	7.4 (4.0)	6.8 (3.2)
TOR-BSST, failed (%)	126	122 (96.8)	45 (100)	77 (95.1)	0.30	81	41 (95.3)	36 (94.7)
Feeding route (%)	126				0.14	81	43	38
Oral, normal diet		7 (5.6)	5 (11.1)	2 (2.5)			2 (4.7)	0 (0)
Oral, soft diet		36 (28.6)	9 (20.0)	27 (33.3)			13 (30.2)	14 (36.8)
Nasogastric		70 (55.6)	27 (60.0)	43 (53.1)			25 (58.1)	18 (47.4)
PEG		2 (1.6)	0 (0)	2 (2.5)			2 (4.7)	0 (0)
Other		11 (8.7)	4 (8.9)	7 (8.6)			1 (2.3)	6 (15.8)
Weight (kg)	126	73.0 (16.1)	74.1 (16.5)	72.4 (15.9)	0.58	81	71.5 (14.8)	73.4 (17.2)
Body mass index (kg/m^2^)	122	25.6 (4.9)	25.7 (4.9)	25.5 (4.9)	0.88	77	25.8 (4.3)	25.3 (5.5)
Mid-arm circumference (cm)	125	28.5 (3.6)	28.2 (3.4)	28.6 (3.7)	0.61	80	28.3 (3.4)	28.9 (4.0)
Albumin (g/L)	120	3.6 (0.6)	3.6 (0.6)	3.7 (0.5)	0.44	77	3.7 (0.6)	3.6 (0.5)
Chest infection (%)	126	5 (4.0)	3 (6.7)	2 (2.5)	0.35	81	1 (2.3)	1 (2.6)

CT: computed tomography; DSRS: dysphagia severity rating scale; ICH: intracerebral haemorrhage; NIHSS: National Institutes of Health Stroke Scale; PEG: percutaneous endoscopic gastrostomy; TACS: total anterior circulation syndrome; PACS: partial anterior circulating syndrome; POCS: posterior communicating syndrome; TOR-BSST: Toronto Bedside Swallowing Screening Test. PES group associated with lower mRS (*P* = 0.032) and higher Barthel Index (*P* = 0.032).

**Table 2:** VFSS measures at baseline for 5 ml mode swallow. Data are number (%) or mean (SD); comparison by Chi-Square (Exact) Test/Fisher's Exact Test or Welch's *T*-Test.

5 ml measures-mode swallow	*N*	All	PES	Sham
PAS, modal bolus	72	4.4 (2.9)	4.3 (3.1) [38]	4.5 (2.6) [34]
PAS, mean all boli	72	4.5 (1.8)	4.4 (1.9) [38]	4.7 (1.7) [34]
OTT (ms)	40	2.14 (3.39)	2.65 (4.14) [25]	1.30 (1.16) [15]
SRT	62	2.07 (6.55)	2.32 (8.53) [33]	1.77 (3.20) [29]
ILC	56	2.55 (6.87)	2.91 (9.08) [29]	2.16 (3.29) [27]
LVC_rt	57	0.38 (0.15)	0.39 (0.17) [29]	0.38 (0.12) [28]
LCD	56	0.44 (0.21)	0.50 (0.25) [28]	0.39 (0.13) [28]^∗^
PRT	36	0.86 (0.12)	0.89 (0.13) [20]	0.82 (0.10) [16]
PTT	37	3.73 (8.36)	4.29 (10.88) [20]	3.06 (3.96) [17]
UOSD	38	0.62 (0.16)	0.65 (0.17) [20]	0.59 (0.13) [18]
Initiation of pharyngeal swallow (0-4)	69		37	32
Bolus head-ramus		10 (14.5)	5 (13.5)	5 (15.6)
Bolus head-valleculae		12 (17.4)	10 (27.0)	2 (6.3)
Bolus head-laryngeal surface		8 (11.6)	2 (5.4)	6 (18.8)
Bolus head-pyriforms		39 (56.5)	20 (54.1)	19 (59.4)
No visible initiation		0 (0)	0 (0)	0 (0)
Oral residue (0-4)	56		28	28
Complete clearance		0 (0)	0 (0)	0 (0)
Trace residue		12 (21.4)	9 (32.1)	3 (10.7)
Residue collection		42 (75.0)	18 (64.3)	24 (85.7)
Majority bolus remaining		1 (1.8)	1 (3.6)	0 (0)
Minimal/no clearance		1 (1.8)	0 (0)	1 (3.6)
Pharyngeal residue (0-4)	63		34	29
Complete clearance		4 (6.3)	3 (8.8)	1 (3.4)
Trace residue		19 (30.2)	11 (32.4)	8 (27.6)
Residue collection		39 (61.9)	19 (55.9)	20 (69.0)
Majority bolus remaining		0 (0)	0 (0)	0 (0)
Minimal/no clearance		1 (1.6)	1 (2.9)	0 (0)
No. of swallows to clear 5 ml	72	1.1 (1.1)	1.3 (1.1) [38]	0.8 (1.0) [34]^∗^

LCD was shorter in the sham group (*P* = 0.039), and fewer clearing swallows were seen in the sham group (*P* = 0.048).

**Table 3:** Comparison of PAS, timing, and clearance measures for 5 ml mode swallow, by change score from baseline to two weeks, using Independent Samples *T*-Test (continuous variables) and Mann–Whitney *U* Test (ordinal variables).

5 ml measures–mode swallow	*N*	PES Mean (SD)	*N*	No PES Mean (SD)	Difference (95% CI)	*P* value
PAS, modal bolus	38	-1.45 (3.06)	33	-0.85 (2.54)	-0.60 (-1.93, 0.73)	0.37
PAS, mean all boli	38	-0.99 (1.66)	34	-0.95 (1.58)	-0.04 (-0.80, 0.72)	0.91
OTT	20	-0.64 (1.83)	9	-0.51 (1.37)	-0.13 (-1.41, 1.14)	0.83
SRT	28	-1.98 (8.94)	27	-1.05 (3.39)	-0.92 (-4.60, 2.76)	0.62
ILC	24	-2.04 (9.73)	24	-1.17 (3.55)	-0.87 (-5.19, 3.45)	0.68
LVC_rt	25	-0.07 (0.15)	25	-0.01 (0.19)	-0.07 (-0.17, 0.03)	0.18
LCD	22	0.04 (0.18))	25	-0.00 (0.19)	0.04 (-0.07, 0.15)	0.43
PRT	15	0.01 (0.16)	9	0.01 (0.08)	-0.00 (-0.10, 0.10)	0.97
PTT	15	-3.66 (12.12)	10	-1.49 (5.26)	-2.17 (-9.55, 5.21)	0.55
UOSD	15	0.03 (0.15)	10	0.00 (0.09)	0.03 (-0.07, 0.13)	0.58
Initiation of pharyngeal swallow (0-4)	36	0 [2]	32	0 [1]	-0.5 (-1,0)	0.52
Ramus		8 (22.2)		5 (15.6)		
Valleculae		6 (16.7)		2 (6.3)		
Laryngeal surface		5 (13.9)		3 (9.4)		
Pyriforms		17 (47.2)		22 (68.8)		
No visible initiation		0 (0.0)				
Oral residue (0-4)	22	0 [0]	23	0 [0]	-0.5 (-1,0)	0.64
Complete clearance		0 (0.0)		0 (0.0)		
Trace residue		7 (31.8)		5 (21.7)		
Residue collection		14 (63.6)		18 (78.3)		
Majority bolus remaining		1 (4.5)		0 (0.0)		
Minimal/no clearance		0 (0.0)		0.(0.0)		
Pharyngeal residue (0-4)	31	0 [0]	29	0 [1]	0 (-1,0)	0.53
Complete clearance		2 (6.5)		0 (0.0)		
Trace residue		11 (35.5)		14 (48.3)		
Residue collection		18 (58.1)		15 (51.7)		
Majority bolus remaining		0 (0.0)		0.(0.0)		
Minimal/no clearance		0 (0.0)		0 (0.0)		
No. of swallows to clear 5 ml	38	0.11 (1.39)	33	0.12 (1.22)	-0.02 (-0.63, 0.60)	0.96

**Table 4:** VFSS measures at baseline for 50 ml bolus (comparison by Chi-square/Fisher's Exact Test or Welch's *T*-Test (unpooled test).

50 ml measures at baseline	N	All	PES	Sham
PAS, 50 ml worst	49	6.7 (1.8)	6.6 (1.7) [25]	6.8 (2.0) [24]
PAS, 50 ml mean	49	3.6 (1.9)	3.3 (1.8) [25]	3.9 (1.9) [24]
No. swallows to clear	49	7.8 (5.0)	8.0 (5.5) [25]	7.5 (4.6) [24]
Initiation of pharyngeal swallow	45		22	23
Bolus head-ramus		3 (6.7)	3 (13.6)	0 (0.0)
Bolus head-valleculae		4 (8.9)	2 (9.1)	2 (8.7)
Bolus head-laryngeal surface		5 (11.1)	3 (13.6)	2 (8.7)
Bolus head-pyriforms		33 (73.3)	14 (63.6)	19 (82.6)
No visible initiation		0 (0)	0 (0.0)	0 (0.0)
Oral residue (0-4)	40		22	18
Complete clearance		0 (0)	0 (0.0)	0 (0.0)
Trace residue		1 (2.5)	1 (4.5)	0 (0.0)
Residue collection		37 (92.5)	20 (90.9)	17 (94.4)
Majority bolus remaining		1 (2.5)	1 (4.5)	0 (0.0)
Minimal/no clearance		1 (2.5)	0 (0.0)	1 (5.6)
Pharyngeal residue (0-4)	43		23	20
Complete clearance		0 (0)	0 (0.0)	0 (0.0)
Trace residue		10 (23.3)	7 (30.4)	3 (15.0)
Residue collection		31 (72.1)	14 (60.9)	17 (85.0)
Majority bolus remaining		1 (2.3)	1 (4.3)	0 (0.0)
Minimal/no clearance		1 (2.3)	1 (4.3)	0 (0.0)

**Table 5:** Comparison of PAS, timing, and clearance measures for 50 ml worst swallow, by change score from baseline to two weeks, using Independent Samples *T*-Test (continuous variables) and Mann–Whitney *U* Test (ordinal variables).

50 ml measures at 2 weeks	*N*	PES Mean (SD)	*N*	No PES Mean (SD)	Difference/95% (CI)	*P* value
PAS, 50 ml worst	24	-1.04 (2.73)	22	-0.95 (3.03)	-0.09 (-1.81, 1.63)	0.92
PAS, 50 ml mean	24	-0.42 (1.93)	22	-0.64 (1.59)	0.22 (-0.83, 1.27)	0.68
No. swallows to clear	24	-1.17 (4.30)	22	-1.27 (4.92)	0.11 (-2.65, 2.87)	0.94
Initiation of pharyngeal swallow (0-4)	21		20			0.58
Bolus head-ramus		4 (19.0)		1 (5.0)		
Bolus head-valleculae		0 (0.0)		0 (0.0)		
Bolus head-laryngeal surface		3 (14.3)		5 (25.0)		
Bolus head-pyriforms		14 (66.7)		14 (70.0)		
No visible initiation		0 (0.0)		0 (0.0)		
Oral residue (0-4)	19		15			0.26
Complete clearance		0 (0.0)		0 (0.0)		
Trace residue		0 (0.0)		2 (13.3)		
Residue collection		18 (94.7)		13 (86.7)		
Majority bolus remaining		0 (0.0)		0 (0.0)		
Minimal/no clearance		1 (5.3)		0 (0.0)		
Pharyngeal residue (0-4)	21		17			0.35
Complete clearance		0 (0.0)		0 (0.0)		
Trace residue		7 (33.3)		2 (11.8)		
Residue collection		14 (66.7)		15 (88.2)		
Majority bolus remaining		0 (0.0)		0 (0.0)		
Minimal/no clearance		0 (0.0)		0 (0.0)		

## Data Availability

Data is not available for sharing due to commercial sensitivity.

## Supplementary Materials

Supplementary Materials Supplementary Table 1: summary of measures carried out on mode 5 ml bolus and worst 50 ml bolus. Supplementary Table 2: operational definitions of timing measures. Supplementary Figure 1: distribution of frame rates (*N* = 126) at 2 weeks. Supplementary Figure 2: percentage of included VFSS files (*N* = 81) entered into study at each of 15 hospital sites at 2 weeks. Supplementary Figure 3: longitudinal changes for PAS scores at baseline and two weeks, comparison by Wilcoxon Signed Ranks Test, data are mean (standard deviation). Supplementary Figure 4: longitudinal changes for timing measures (speed and duration) at baseline and two weeks, comparison by Wilcoxon Signed Ranks Test, data are mean (standard deviation). Supplementary Figure 5: longitudinal changes for initiation of pharyngeal swallow and oral and pharyngeal residue, comparison by Wilcoxon Signed Ranks Test.
