# Supplementary material for: Effects of Pharyngeal Electrical Stimulation on Swallow Timings, Clearance and Safety in Post-Stroke Dysphagia: Analysis from the Swallowing Treatment Using Electrical Pharyngeal Stimulation (STEPS) Trial
Source: Stroke Res Treat. 2021 Jun 7;2021:5520657. doi: 10.1155/2021/5520657 (PMC8205591; doi:10.1155/2021/5520657)
Supplement: Supplementary Materials — Supplementary Table 1: summary of measures carried out on mode 5 ml bolus and worst 50 ml bolus. Supplementary Table 2: operational definitions of timing measures. Supplementary Figure 1: distribution of frame rates (N = 126) at 2 weeks. Supplementary Figure 2: percentage of included VFSS files (N = 81) entered into study at each of 15 hospital sites at 2 weeks. Supplementary Figure 3: longitudinal changes for PAS scores at baseline and two weeks, comparison by Wilcoxon Signed Ranks Test, data are mean (standard deviation). Supplementary Figure 4: longitudinal changes for timing measures (speed and duration) at baseline and two weeks, comparison by Wilcoxon Signed Ranks Test, data are mean (standard deviation). Supplementary Figure 5: longitudinal changes for initiation of pharyngeal swallow and oral and pharyngeal residue, comparison by Wilcoxon Signed Ranks Test. [file 5520657.f1.docx]

**SUPPLEMENTARY FILES**

**Effects of pharyngeal electrical stimulation on swallow timings, clearance and safety in Post-Stroke Dysphagia: Analysis from the Swallowing Treatment using Electrical Pharyngeal Stimulation (STEPS) Trial**

Lisa F Everton MPhil;^1,2^ Jacqueline K Benfield MSc;^3^ Emilia Michou PhD;^4,5^ Shaheen Hamdy PhD FRCP;^4^ Philip M Bath DSc FMedSci ^1,6^

1. Stroke Trials Unit, Mental Health and Clinical Neuroscience, University of Nottingham, Nottingham, UK

2. Speech and Language Therapy, Nottinghamshire Healthcare NHS Foundation Trust, Nottingham, UK

3. Vascular Medicine, Division of Medical Sciences and GEM, University of Nottingham, Royal Derby Hospital Centre, Derby, UK

4. GI Sciences, Division of Diabetes, Endocrinology and Gastroenterology, School of Medicine Sciences, University of Manchester and the Manchester Academic Health Sciences Centre, UK

5. Speech and Language Therapy Department, School of Rehabilitation Sciences, University of Patras, Patras, Greece

6. Stroke, Nottingham University Hospitals NHS Trust, Nottingham, UK

# **Supplementary Table 1.** Summary of measures carried out on mode 5ml bolus and worst 50ml bolus

| **Measure** | **Component** |
| --- | --- |
| 5ml scoring design | Mode bolus |
| 5ml timing measures | Global Oral Transit Time (gOTT) |
|  | Stage Transition Duration (STD) |
|  | Initiation of pharyngeal swallow (MBSImP, component 6) |
|  | Initiation of Laryngeal Closure (ILC) |
|  | Laryngeal Vestibule Closure- reaction time (LVCrt) |
|  | Laryngeal Closure Duration (LCD) |
|  | Pharyngeal Response Time (PRT) |
|  | Pharyngeal Transit Time (PTT) |
|  | Upper Oesophageal Sphincter Duration (UOSD) |
| 5ml clearance measures | Oral residue score 0-4 (MBSImP, component 5) |
|  | Pharyngeal residue 0-4 (MBSImP, component 16) |
|  | Number of swallows to clear 5ml bolus |
| 50ml scoring design | Worst bolus |
| 50ml timing measures | Swallow Response Time by location (MBSImP, component 6) |
| 50ml clearance measures | Oral residue (MBSImP, component 5) |
|  | Pharyngeal residue (MBSImP, component 6) |
|  | Number of swallows to clear 50ml bolus |

# **Supplementary Table 2.** Operational definitions of timing measures

| Global oral transit time (gOTT, seconds) | The interval between the frame showing onset of manipulation of the bolus by the tongue in the oral cavity and the head of the bolus reaching the angle of the ramus of the mandible |
| --- | --- |
| Stage transition duration (STD, seconds) | The interval between the frame showing the head of the bolus reaching the angle of the ramus of the mandible and the frame showing onset of anterior-superior hyoid movement, associated with a swallow ^1^ |
| Initiation of laryngeal closure (ILC, seconds) | The interval between the frame showing the head of the bolus reaching the angle of the ramus of the mandible and the frame showing contact of the arytenoids with base of the epiglottis ^1, 2^ |
| Laryngeal vestibule closure-reaction time (LVCrt, seconds) | The interval between the frame showing onset of anterior-superior hyoid movement, associated with a swallow and the frame showing contact of the arytenoids with base of the epiglottis ^3^ |
| Laryngeal closure duration (LCD, seconds) | The interval from the frame showing contact of the arytenoids with base of the epiglottis (airway closure) to the last frame showing this contact has discontinued (airway opening) ^4^ |
| Pharyngeal response time (PRT, seconds) | The interval from the frame showing onset of initiation of laryngeal elevation to the frame showing the tail of the bolus passing into the upper oesophageal sphincter (UOS) ^4^ |
| Pharyngeal transit time (PTT, seconds) | The interval from the frame showing the head of the bolus reaching the angle of the ramus of the mandible to the frame showing the tail of the bolus passing into the UOS ^5^ |
| Upper oesophageal sphincter duration (UOSD, seconds) | The interval from first opening of the UOS (as signified by a column of air ^6^ or of contrast entering the narrowest part of the UOS) ^7^ to the frame showing the tail of the bolus passing into the UOS ^5^ |
| Initiation of pharyngeal swallow (IPS, range 0-4) ^8^ | Location of bolus head when initiation of pharyngeal swallow is triggered. 0: bolus head at posterior angle of ramus; 1: bolus head in valleculae; 2: bolus head at posterior laryngeal surface of epiglottis; 3: bolus head in pyriforms; 4: no visible initiation at any location |
| Oral residue (OR, range 0-4) ^8^ | Residue in oral cavity. 0: complete oral clearance; 1: trace residue lining oral structures; 2: residue collection on oral structures; 3: majority of bolus remaining; 4: minimal to no clearance. |
| Pharyngeal residue (PR, range 0-4) ^8^ | Residue in pharynx. 0: complete pharyngeal clearance; 1: trace residue within or on pharyngeal structures; 2: collection of residue within or on pharyngeal structures; 3: majority of contrast within or on pharyngeal structures; 4: minimal to no pharyngeal clearance. |

# **Supplementary Figure 1**. Distribution of frame rates (N=126) at 2 weeks


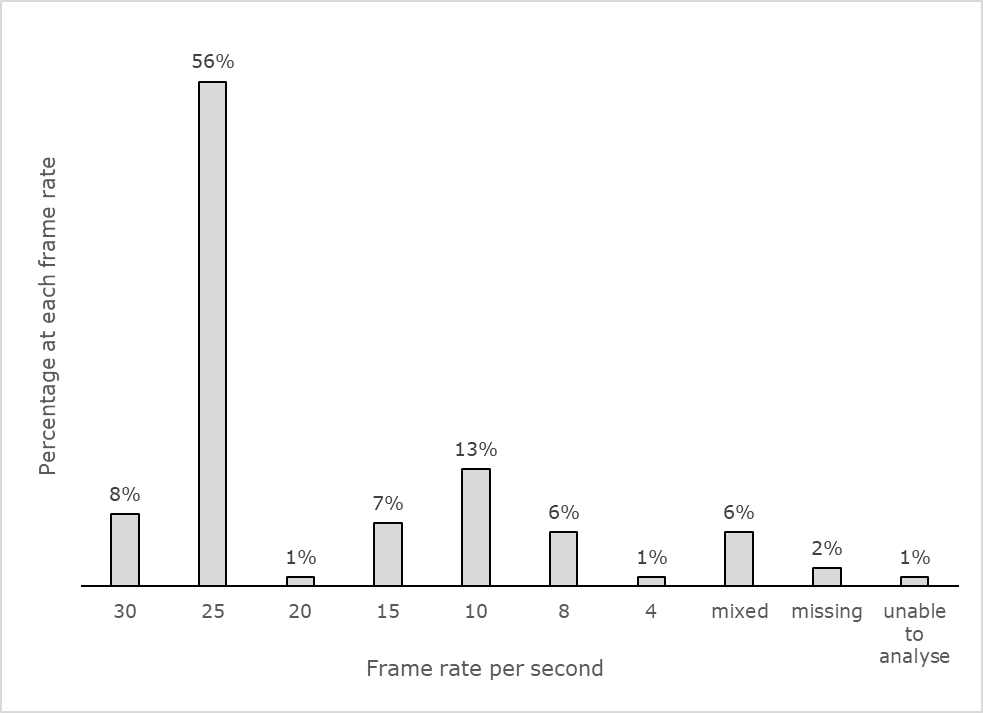


# **Supplementary Figure 2.** Percentage of included VFSS files (N=81) entered into study at each of 15 hospital sites at 2 weeks


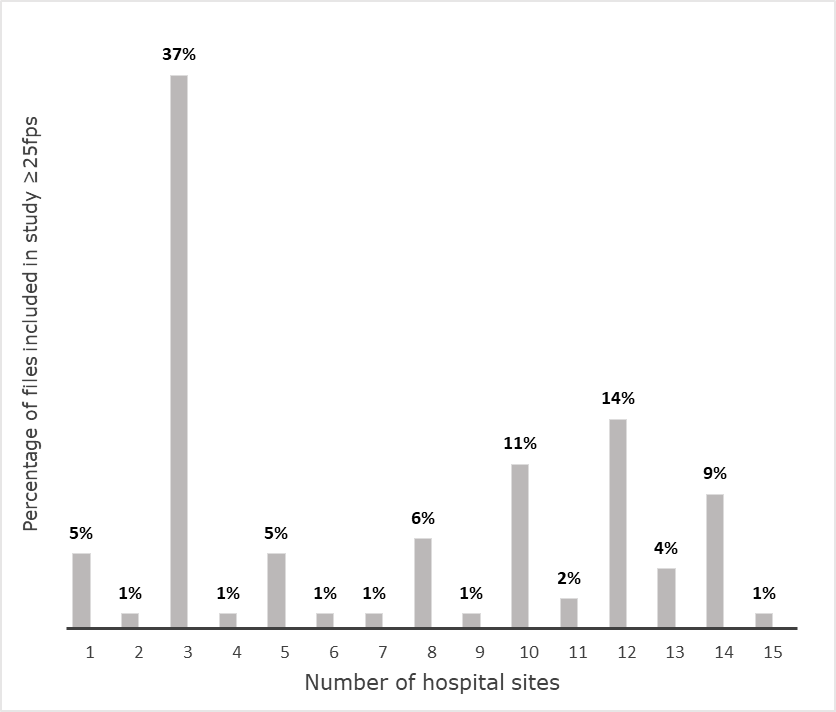


# **Supplementary Figure 3.** Longitudinal changes for PAS scores at baseline and two weeks, comparison by Wilcoxon Signed Ranks Test, data are mean (standard deviation), PAS score range 1-8

* Denotes significant at p<0.05, ** denotes highly significant (P < 0.001)

# **Supplementary Figure 4.** Longitudinal changes for timing measures (speed and duration) at baseline and two weeks, comparison by Wilcoxon Signed Ranks Test, data are mean (standard deviation), mean timings at both timepoints are in seconds

* Denotes significant at p<0.05

# **Supplementary figure 5.** Longitudinal changes for initiation of pharyngeal swallow and oral and pharyngeal residue, comparison by Wilcoxon Signed Ranks Test, data are 0-4 as per MBSImP

**
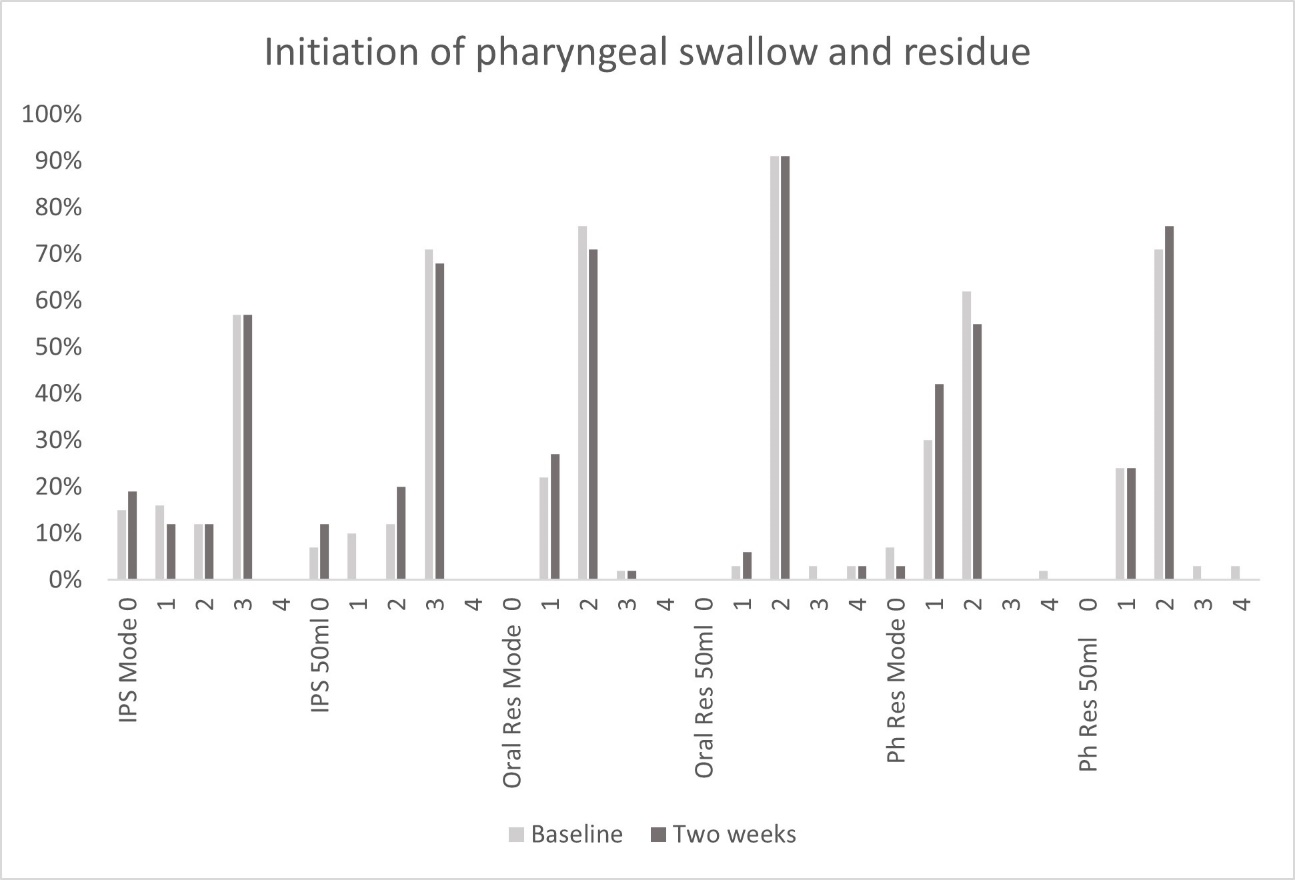
**

# REFERENCES

1. Robbins J, Hamilton JW, Lof GL, et al. Oropharyngeal swallowing in normal adults of different ages. *Gastroenterology* 1992; 103: 823-829. DOI: 10.1016/0016-5085(92)90013-o.

2. Rademaker AW, Pauloski BR, Logemann JA, et al. Oropharyngeal swallow efficiency as a representative measure of swallowing function. *J Speech Hear Res* 1994; 37: 314-325. DOI: 10.1044/jshr.3702.314.

3. Macrae P, Anderson C and Humbert I. Mechanisms of airway protection during chin-down swallowing. *J Speech Lang Hear Res* 2014; 57: 1251-1258. DOI: 10.1044/2014_JSLHR-S-13-0188.

4. Rademaker AW, Pauloski BR, Colangelo LA, et al. Age and volume effects on liquid swallowing function in normal women. *J Speech Lang Hear Res* 1998; 41: 275-284. DOI: 10.1044/jslhr.4102.275.

5. Power ML, Fraser CH, Hobson A, et al. Evaluating oral stimulation as a treatment for dysphagia after stroke. *Dysphagia* 2006; 21: 49-55. DOI: 10.1007/s00455-005-9009-0.

6. Jacob P, Kahrilas PJ, Logemann JA, et al. Upper esophageal sphincter opening and modulation during swallowing. *Gastroenterology* 1989; 97: 1469-1478. DOI: 10.1016/0016-5085(89)90391-0.

7. Kendall KA, McKenzie S, Leonard RJ, et al. Timing of events in normal swallowing: a videofluoroscopic study. *Dysphagia* 2000; 15: 74-83. DOI: 10.1007/s004550010004.

8. Martin-Harris B, Brodsky MB, Michel Y, et al. MBS measurement tool for swallow impairment--MBSImp: establishing a standard. *Dysphagia* 2008; 23: 392-405. 2008/10/15. DOI: 10.1007/s00455-008-9185-9.
